# On the Treatment of Cholera with Strychnia

**Published:** 1855-11

**Authors:** A. P. Merrill

**Affiliations:** Professor of Principles and Practice of Medicine in the Memphis Medical College


*On the Treatment of Cholera with Strychnia.* By A. P. Merrill, M. D., Professor of Principles and Practice of Medicine in the Memphis Medical College.

After the vast variety of plans and remedies which have been proposed for the successful treatment of cholera, and the failure of the profession, under any system of treatment to cure, as a rule, more than one-half the cases that occur, showing scarcely greater success at the present time than when the disease first invaded Europe and America, it is with diffidence that we venture to make even a single suggestion upon the subject; and the more, as the moderate number of cases we have met with in this city, where the disease has prevailed only sporadically, has not given us a very full opportunity to put the practice we are about to propose, to an ample test. For several years past it has been our practice to rely largely upon strychnia in the treatment of diarrhea, particularly in its incipient stage; and we have found it, both with white people and with plantation slaves, a most efficient and convenient remedy in this disease. It has proved especially useful, in the relief of those sudden derangements of the digestive organs, consequent upon imprudence in eating, whether dependent upon an excessive quantity of food, or upon such as is of an indigestible character; leading, in the first place, to sensations of fullness and oppression in the epigastrium, then to commotion and more or less pain in the intestinal canal, followed by copious alvine discharges and sometimes by exhausting serous stools.

These symptoms, so readily relieved by strychnia, simulate cholera to a sufficient extent to justify the expectation, that a similar treatment might be applied to both. Accordingly we have occasionally experimented, upon well developed cases of cholera, with moderate doses of strychnia, as an accompaniment to other remedies, and sometimes with apparently useful results. In the treatment of the premonitory symptoms, choleraic diarrhea, we have rarely ever prescribed any other remedy; and this stage of the disease being almost identical with the diarrhea above described, we have found this prescription equally effectual in both. Indeed its remedial action is so prompt and decided in such cases, that more than one or two doses are seldom necessary.

These and other observations upon the remedial efficacy of this remedy, as well as favorable reports from other quarters, led us to give it a more thorough trial in cases of cholera, than we had hitherto done. At first we alternated it with large doses of calomel, and successfully, but without the prompt suspension of the alvine discharges, which it accomplishes in diarrhea. This led us to suspect, that the cathartic operation of the calomel counteracted, to some extent, the remedial power of the strychnia. Subsequently, therefore, we used the strychnia alone, giving no other medicine while the cholera symptoms prevailed, but following the subsidence of cholera with a few small doses of calomel to excite the secretions, and produce moderate catharsis. Our experience with this treatment, although somewhat limited, has been sufficient to convince us, that strychnia possesses antidotal powers to the action of the cholera poison, to a much greater extent than any other remedy in use, and to an extent to render it of great value in the treatment of the disease.

The first effect of strychnia used in this way is, to check or wholly suspend the rice-water discharges from the bowels. The most striking relief however, afforded by it, arises from its influence over the capillary circulation; and it is only after its effects over this function are perceptible, that its remedial power becomes decided. That degree of capillary congestion which causes the purple skin, and enables it to retain the marks occasioned by pressure upon the surface for a considerable length of time after the pressure is removed, is gradually relieved as the patient comes under the operation of the remedy, accompanied by an equable and wholesome glow of warmth over the whole body. Cramps in the limbs subside as soon as this influence is fully exerted, and the pulse improves in fullness and strength. Unless the toxical effects of the remedy follow, there is also an abatement of thirst, and a return of warmth to the tongue and mouth. Huskiness of the voice gradually subsides and the patient is relieved of the extreme prostration, which constitutes one of the most distressing symptoms of cholera.

These are remedial influences scarcely less decided and remarkable than those which follow the use of quinia in periodic fever. But it is no small disadvantage, that the toxical effects of strychnia are so easily produced, and are of such serious character, as very much to lessen the value and importance of the remedy, unless some means are discovered for obviating the evil. The antidotal power of camphor does not appear yet to be well established, and it is doubtful whether any other has been discovered which promises even so much as this. The toxical effects are the more to be feared because of the difficulty in ascertaining, while frequent vomiting continues the exact quantity of the medicine retained, or the quantity which may lie dormant in the stomach and bowels, from a temporary suspension of the assimilative powers, and thus endanger the patient from its cumulative action whenever assimilation is restored.

For the cure of diarrhea, whether constituting the early stage of cholera or not, we have generally given strychnia in crystals, made up into pills, with gum arabic and simple sirup, and in doses of one-twentieth to one-tenth of a grain. The stomach in this case, not having lost its digestive powers, and the symptoms being less urgent than in cholera, it is not often necessary to repeat the dose, except sometimes at long intervals, and thus the danger of cumulation is easily obviated. But when, in severe cases of cholera, it becomes necessary to repeat the dose every hour or two, we are not so certain of this ready assimilation of the remedy, or even of the immediate solution of the pills in the fluids of the stomach. To guard against this difficulty, we have given the remedy in solution, using as a solvent two drams of acetic acid and one ounce of alcohol to the grain of crystallized strychnia. It is a serious objection to this formula, that it is apt to prove offensive to the stomach from its extreme bitterness, causing frequent vomiting, and thus rendering it uncertain how much is retained.

Several other physicians in this city have used the crystals of strychnia in the treatment of cholera, pretty much in the same way; and some are of opinion that it is the only remedy upon which reliance can be placed, for the relief of the urgent symptoms of that fatal disease. Nor do they, in general, apprehend very serious difficulties from the toxical effects to which we have referred, as the remedy may be suspended, or given less frequently, as soon as its curative action becomes apparent, or as soon as it begins to exhibit its peculiar influence over the capillary engorgements. In a disease which, in a majority of cases, reaches what may be considered its fatal stage, before the physician is called in, something may be risked upon the toxical effects of any remedy which gives much promise of success in the treatment. Still, it would be but poor consolation to know that our patient has been relieved of cholera, only to die under the toxical effects of the medicine employed, although it may have been the only one capable of suspending the disease. But it must be remembered, that we are subject to the same difficulties in the use of mercury, arsenic, and various other articles, which, being powerful for good, are necessarily powerful for evil also.—*Memphis Medical Recorder.*

